# SciDBMaker: new software for computer-aided design of specialized biological databases

**DOI:** 10.1186/1471-2105-9-121

**Published:** 2008-02-25

**Authors:** Riadh Hammami, Abdelmajid Zouhir, Karim Naghmouchi, Jeannette Ben Hamida, Ismail Fliss

**Affiliations:** 1 Unité de Protéomie Fonctionnelle & Biopréservation Alimentaire, Institut Supérieur des Sciences Biologiques Appliquées de Tunis, Université El Manar, Tunisie; 2 Agriculture and Agri-Food Canada, Lethbridge Research Centre, Lethbridge, Alberta, T1J 4B1 Canada; 3 Institut des Nutraceutiques et des Aliments Fonctionnels (INAF), Université Laval, Québec, Canada

## Abstract

**Background:**

The exponential growth of research in molecular biology has brought concomitant proliferation of databases for stocking its findings. A variety of protein sequence databases exist. While all of these strive for completeness, the range of user interests is often beyond their scope. Large databases covering a broad range of domains tend to offer less detailed information than smaller, more specialized resources, often creating a need to combine data from many sources in order to obtain a complete picture. Scientific researchers are continually developing new specific databases to enhance their understanding of biological processes.

**Description:**

In this article, we present the implementation of a new tool for protein data analysis. With its easy-to-use user interface, this software provides the opportunity to build more specialized protein databases from a universal protein sequence database such as Swiss-Prot. A family of proteins known as bacteriocins is analyzed as 'proof of concept'.

**Conclusion:**

SciDBMaker is stand-alone software that allows the extraction of protein data from the Swiss-Prot database, sequence analysis comprising physicochemical profile calculations, homologous sequences search, multiple sequence alignments and the building of new and more specialized databases. It compiles information with relative ease, updates and compares various data relevant to a given protein family and could solve the problem of dispersed biological search results.

## Background

The exponential growth of molecular biology research in recent decades has brought concomitant growth in the number and size of databases used to interpret experimental findings. For example, UniProtKB/Swiss-Prot release 53.2, dated 26-06-07, contains 272,212 sequence entries comprising 99,940,143 amino acids, abstracted from 157,086 references [1]. A variety of protein sequence databases exist, ranging from simple sequence repositories to expertly curated universal databases that cover all species and in which the original sequence data are enhanced by manual addition of further information in each sequence record [2]. While all of these strive for completeness, the range of user interests is often beyond their scope. This may reflect the user's wish to combine different types of information or the inability of a single resource to contain the complete details of every relevant experiment. In addition, large databases with broad domains tend to offer less detailed information than smaller, more specialized, resources, with the result that data from many resources may need to be combined to provide a complete picture. There is a clear need to gather, filter and critically evaluate this mass of information so that it can be used with greater efficiency. Since scientists are continually developing new specific databases to enhance their understanding of biological processes, we created SciDBMaker to provide a tool for easy building of new specialized protein knowledge bases. This paper describes the development of new stand-alone software, ***Sci****entific ****D****ata****B****ase ****M****aker*, for protein data analysis with online and/or off-line access. The software interface allows successive steps for sequence manipulation, starting from user sequence search and homologous sequence retrieval from the SwissProt databank, followed by physicochemical profile calculations, multiple sequence alignments, phylogenic tree visualization and culminating in database export/building. All steps are performed in an interactive manner. Physical and chemical parameters, rarely found in public databases, provide a helpful tool for the analysis of a set of proteins and their calculation is achieved in a direct and interactive manner, with off-line access. SciDBMaker also processes a great number of sequences simultaneously.

## Implementation

### Swiss-Prot format

The Swiss-Prot format has been described previously in reference [3].

### Physicochemical profiles

Protein families may be analyzed with the help of physicochemical profiles such as amino acid composition (acidic, basic, hydrophobic, polar, absent and common amino acids), atomic composition, molecular weight [4], theoretical pI [4,5], extinction coefficient [6], absorbance at 280 nm, estimated half-life in mammalian cells, yeast and *E. coli *[7,8], instability index [9], aliphatic index [10], grand average of hydropathicity (GRAVY) [11] and protein-binding potential (Boman index) [12].

### Integrated tools

The European Bioinformatics Institute provides the Dbfetch tool for easy retrieval of entries from various databases [13]. Entries may be imported online into SciDBMaker from the SwissProt database using Dbfetch. To find similar sequences, the containers can be queried with either proteins from the SwissProt database or user-imported sequences, using the BLAST algorithm [14]. Multiple sequence alignments (MSA) are an essential tool for predicting protein structure and function prediction, phylogenic inference and other common tasks in sequence analysis. To date, CLUSTALW is still the most popular alignment tool. Since it is the method of choice for biologists, CLUSTALW [15] was included in SciDBMaker for multiple sequence alignments. Generated trees may be easily viewed using phylogenic tree visualization software such as TREEVIEW [16].

### Hardware and software specifications

The executable version of the SciDBMaker software can be installed and run on a standard PC platform with a Windows operating system. The software development was done using Windows XP and tested with success on all platforms, including Win 98, Win XP and Win vista. The source code was written in Microsoft Visual Basic .NET (2005). The environment is based upon the .NET Framework library v2.0.

## Results & discussion

### Program description

**Figure 1:**
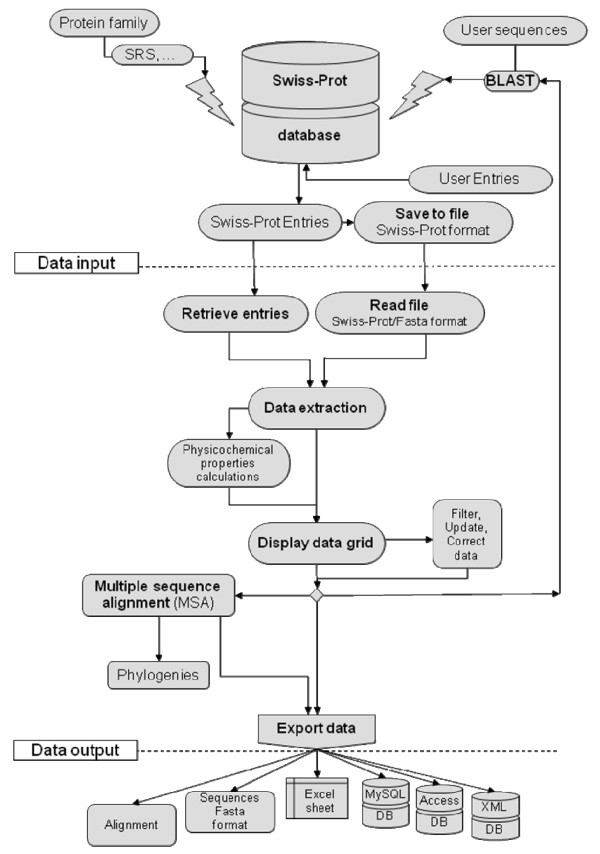
Workflow diagram.

**Figure 2:**
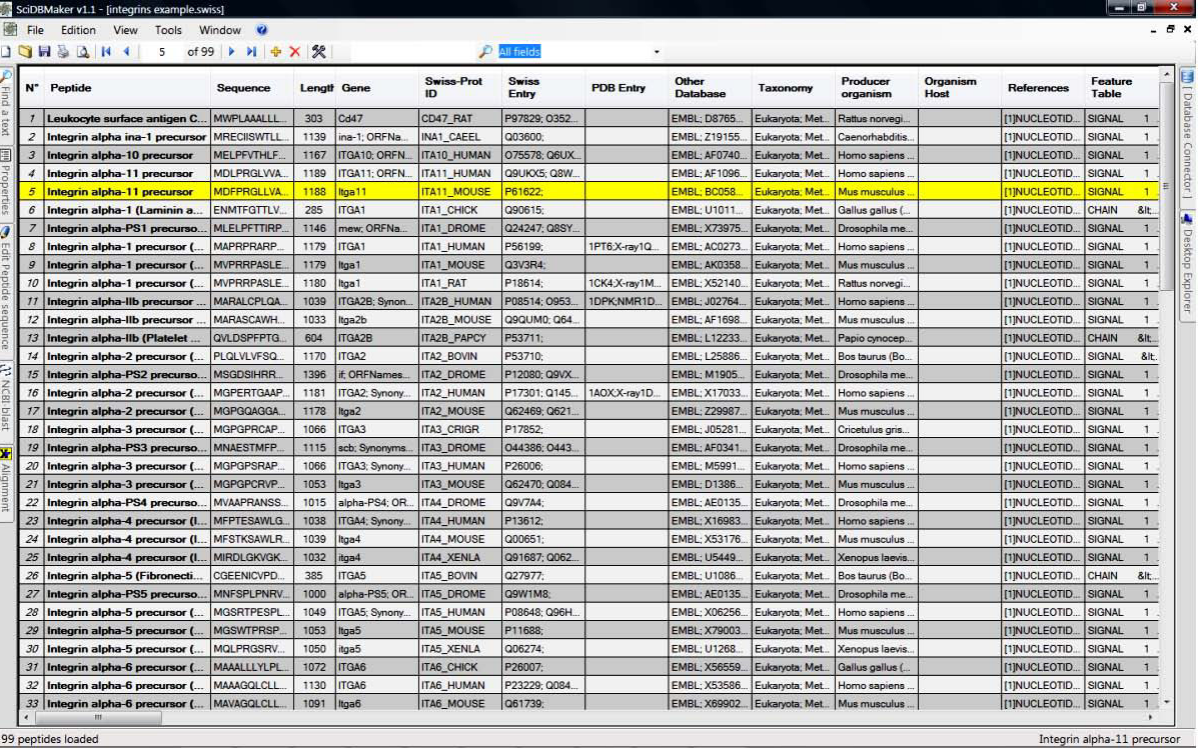
User interface.

**Figure 3:**
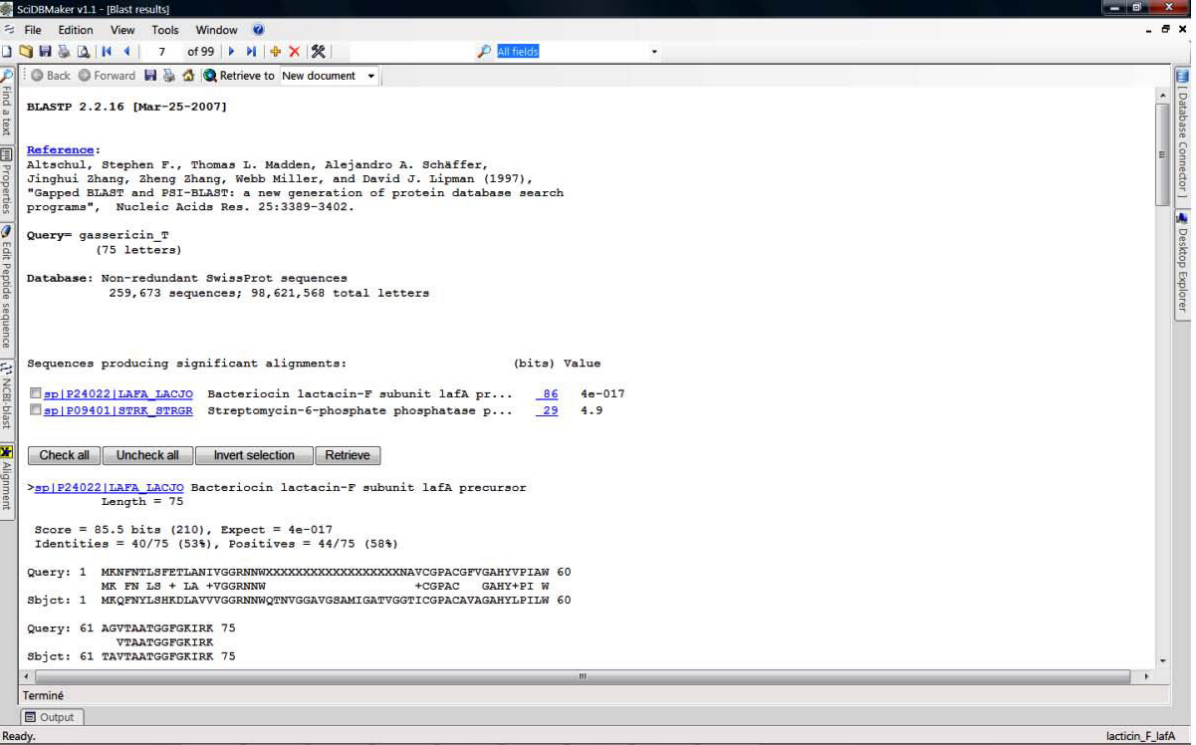
A typical Blast result window.

**Figure 4:**
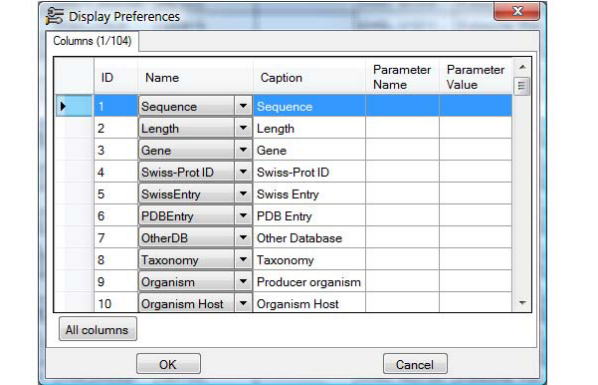
Preference display columns.

The workflow diagram shown in Figure 1 and the following discussion illustrate how the tool works. Figure 2 illustrates a typical user interface of the program. Users may open files in Fasta or Swiss-Prot format, or import sequence entries from the Swiss-Prot database. Users may also use their own sequences, search for homologous sequences entries in Swiss-Prot database using BLAST algorithm and load selected entries into SciDBMaker (Fig. 3). The program will automatically extract available information in Swiss-Prot entries and calculate physicochemical profiles for loaded proteins. Users may also choose the information to be extracted and the properties to be calculated, as shown in Figure 4. The interface allows users to filter, search, add, remove and update data rows as required. An intuitive interface allows BLAST selection of all user sequences. Similarly, sequences may be aligned using the multiple alignment program ClustalW. Resulting trees may be shown using the phylogenic tree visualization software TREEVIEW, as proposed by SciDBMaker. As a final step, data may be printed or saved in various file formats. Sequences may be extracted into a Fasta format file. The resulting data grid may be saved as an MS Excel data sheet, as well as database files (XML, MS Access, MySQL).

### Program runs

A database was developed using SciDBMaker. Named BACTIBASE, this database has been previously described in reference [17].

## Conclusion

The stand-alone software SciDBMaker allows the extraction of protein data from the Swiss-Prot database, sequence analysis comprising physicochemical profile calculations, homologous sequence searches, multiple sequence alignments and the building of new and more specialized databases. Programs of this type compile information with relative ease, update and compare various data relevant to a given protein family and could solve the problem of dispersed biological search results. Collection of a multitude of information regarding a given protein family allows the development of more coherent and focused approaches to structure-function relationships, thereby enhancing the development of theoretical concepts in biological sciences.

## Availability and requirements

The program runs on the PC platform with a Windows operating system. The graphical environment needs the .NET Framework library v2.0. This complement is available for free download at the Microsoft website and comes pre-installed in the majority of recent computers. An installation package for SciDBMaker may be obtained from the authors free of charge upon request. The SciDBMaker website is hosted by the Centre de Calcul El Khawarizmi CCK (Tunisia) and is available at . The SciDBMaker software is provided 'as is' with no guarantee or warranty of any kind and is available for all non-commercial use. Any other use of the software requires special permission from the primary author.

## Authors' contributions

RH programmed the software interface, performed the implementation of physicochemical parameters and drafted the manuscript. AZ participated in the design of the study, interacted with RH to carry out the physicochemical data calculation and tested the program. KN tested the program and contributed to the manuscript. JBH oversaw the project and helped define user requirements. IF conceived the study, participated in its design and coordination and helped to draft the manuscript. All authors read and approved the final manuscript.
